# Acute Sensory-Motor Axonal Neuropathy in a 57-Year-Old Male Presenting With Paresthesia and Distal Muscle Weakness

**DOI:** 10.7759/cureus.11301

**Published:** 2020-11-02

**Authors:** Azka Latif, Vikas Kapoor, Akshat Sood, Joseph Thirumalareddy, Abubakar Tauseef

**Affiliations:** 1 Internal Medicine, CHI Creighton University, Omaha, USA; 2 Hospital Medicine, CHI Creighton University, Omaha, USA

**Keywords:** neurologic disorder, paralysis, inflammatory

## Abstract

Guillain‐Barré syndrome (GBS) is a relatively uncommon post-infectious, immune‐mediated neurologic disorder with an incidence of 0.5-2/100,000. It is usually preceded by an infection that evokes an immune response that cross-reacts with peripheral nerve components via molecular mimicry. The presentation of this disorder has several forms, including acute inflammatory demyelinating polyneuropathy (AIDP), acute motor axonal neuropathy (AMAN), acute motor-sensory axonal neuropathy (AMSAN), and Miller Fisher syndrome (MFS). The case we describe is of a 57-year-old male presenting with sensory features followed by symmetrical ascending paralysis and diagnosed with ASMAN, a recently described subtype of GBS, based on neurological and laboratory findings.

## Introduction

Guillain‐Barré Syndrome (GBS) is a relatively uncommon post-infectious, immune‐mediated neurologic disorder with an incidence of 0.5-2/100,000 characterized by the demyelination of spinal nerve roots and peripheral nerves, and presents with a mono-phasic illness with cerebrospinal fluid (CSF) albuminocytological dissociation with partial or complete recovery [1]. GBS is diagnosed on the basis of a clinical evaluation, CSF findings, and the results of nerve conduction studies. In association with supportive care, immunotherapy via intravenous immunoglobulin (IVIG) administration or plasmapheresis, is the standard treatment for GBS [2].

Besides the classic presentation of GBS, clinical variants are based on the types of nerve fibers involved, the predominant mode of fiber injury (demyelinating versus axonal), and the presence of an alteration in consciousness [3]. These variant forms of GBS often do not fulfill existing diagnostic criteria defined for classical GBS [4]. Hadden et al. evaluated the neurophysiology criteria vs recently proposed Rajabally et al. criteria, which classified GBS into a few subcategories, including classical GBS (41, 67%), classic Miller-Fisher Syndrome (MFS) (6, 10%), pharyngeal-cervical-brachial (PCB) (3, 5%), paraparetic GBS (4, 7%), bifacial weakness with paresthesia (3, 5%), acute ophthalmoparesis (AO) (1, 2%), and overlap syndrome (3, 5%): one (2%) with GBS/Bickerstaff brainstem encephalitis overlap and two (3%) with GBS/MFS overlap [5].

## Case presentation

A 57-year-old male, with no significant past medical history, presented to the emergency department with the complaint of bilateral lower extremity numbness and tingling for two days that was gradually progressive and associated with bilateral lower limb weakness for one day. He didn’t report any history of recent gastrointestinal or respiratory tract infections. There was no history of major surgeries, blood transfusions, or intravenous drug abuse prior to the onset of disease and other comorbid situations.

On clinical examination, the patient was afebrile, conscious, and well-oriented to time, place, and person. The blood pressure was normal (120/75 mmHg), pulse rate was normal (82/minute), and respiratory rate was normal (20/minute). On neurological examination, he had an ataxic gait. There was generalized hyporeflexia and decreased power (3/5) in bilateral lower limbs. In view of these clinical findings, a diagnosis of lower motor neuron type paraparesis was made. The initial laboratory workup was unremarkable. Magnetic resonance imaging (MRI) was obtained to rule out a cerebrovascular accident, which did not reveal any acute changes. While being in the hospital, his lower extremity weakness gradually worsened, followed by numbness and tingling sensation in the bilateral upper extremity.

Further workup with CSF is shown in Table 1.

**Table 1:** CSF analysis CSF: cerebrospinal fluid

Appearance	Clear
Color	Colorless
RBCs	15/uL
TLC	2/uL
Glucose	65mg/dl
Proteins	200mg/dl

Albuminocytologic dissociation raised concern for GBS, therefore, he was started on intravenous immune globulins at a dose of 0.4 g/kg per day for a total of five days. Supportive measures, such as the administration of intravenous fluids and nutritional therapy, were also started. Over the course of hospital stay, his neurologic symptoms gradually started to improve. He worked with a physical and occupational therapist and was discharged in two months. On discharge, he was able to walk with assistance.

## Discussion

All patients initially identified as GBS or related disorders can be subclassified as having classical GBS, classic Miller-Fisher Syndrome (MFS), ASMAN, pharyngeal-cervical-brachial (PCB), paraparetic GBS, bifacial weakness with paresthesia, acute ophthalmoparesis (AO), and overlap syndrome [1]. Pure sensory GBS is a very rare sub-type; to date, <10 cases of pure sensory GBS have been reported; thus, the clinical and pathological features of sensory variant GBS are yet to be well-characterized [6].

ASMAN is a recently described subtype of GBS characterized by the acute onset of sensory symptoms, with a loss of deep tendon reflexes and distal weakness. Electrophysiological studies show mildly reduced nerve conduction velocities in axons combined with a marked reduction of muscle action and sensory nerve action potentials.

Several points of this case are remarkable. Patients with AMSAN, like the case described above, usually experience severe symptoms over a short period of time. They also have a prolonged and incomplete phase of recovery despite treatment with immunomodulatory drugs [7]. The therapy of AMAN and AMSAN is based on that of the acute inflammatory demyelinating polyradiculoneuropathy (AIDP), that is, plasma exchange and intravenous immunoglobulin (IVIG) while the benefit of steroids remains ambiguous [8].

## Conclusions

To conclude, in ASMAN, immunomodulating therapy with IVIG may lead to full recovery in selected cases. Although rare, inflammatory polyradiculoneuropathy should be considered in the differential diagnosis of acute flaccid quadriparesis with length-dependent sensory dysfunction.
